# A Clinico-Pathological Multidisciplinary Team Increases the Efficacy of Skin Biopsy and Reduces Clinical Risk in Dermatology

**DOI:** 10.3390/dermatopathology10020023

**Published:** 2023-06-01

**Authors:** Carlo Francesco Tomasini, Andrea Michelerio, Eugenio Isoletta, Stefania Barruscotti, Barbara Wade, Alba Muzzi

**Affiliations:** 1 Department of Clinical-Surgical, Diagnostic and Pediatric Sciences, University of Pavia, 27100 Pavia, Italy; andrea.michelerio01@universitadipavia.it (A.M.);; 2 Dermatology Clinic, Fondazione IRCCS Policlinico San Matteo, 27100 Pavia, Italy; 3 Department of Science of Public Health and Pediatrics, University of Turin, 10126 Turin, Italy; 4 Department of Quality and Risk Management, Fondazione IRCCS Policlinico San Matteo, 27100 Pavia, Italy

**Keywords:** biopsy, dermatology, dermatopathology, risk management, interdisciplinary communication, melanoma, malpractice

## Abstract

A clinical risk is an inherent risk in healthcare processes, including skin biopsy procedures, and may lead to misdiagnoses, increased healthcare costs and potential harm to patients. Indeed, clinical and histopathological data must be integrated if we are to reduce clinical risks and improve diagnostic accuracy in the diagnosis of dermatologic diseases. Although dermopathology services used to be part of a dermatologist’s duty, the recent centralization of these laboratories has caused a loss of expertise and increased both complexity and safety issues. Some countries have implemented clinical-pathological correlation programs aimed at facilitating communication between clinicians and dermatopathologists. However, Italy has regulatory and cultural barriers that make the implementation of these programs difficult. Therefore, an internal analysis was carried out to assess the efficacy and impact that skin biopsy procedures for inflammatory and neoplastic conditions have on the quality of care in our dermatology department. As the analysis evidenced a high number of descriptive pathologic reports and discordant diagnoses, a multidisciplinary group of four dermatologists, four general pathologists and one dermatopathologist was set up. Herein, we present the results of this analysis and project and describe the structure of the multidisciplinary group. We also discuss the pros and cons, possibilities and limitations of our project, including the regulatory barriers of the Italian National Health System.

## 1. Clinical Risk, Patient Experience and Risk Management

Patient safety, i.e., the prevention of harm to patients, is a subset of healthcare quality and is in constant evolution, where risk elimination or reduction is a moral and ethical responsibility of all healthcare professionals [1].

Clinical risk refers to any action that occurs in a healthcare setting that contains a chance of error that may have negative consequences for the patient. It is defined as “the failure to plan and/or execute a sequence of actions that results in the failure to achieve the desired objective, which is not attributable to chance” [2].

Clinical risk (R) expresses both the probability that an error may occur and the potential harm that error might cause to a patient [2,3]. Therefore, risk is a measure of the damage potential of a generic hazard event and is expressed as the product of the probability of occurrence of the event (P) times the severity (magnitude) of the associated damage (D).

R = P × D

The calculation of risk also takes into consideration the ability of the human factor to detect in advance and contain the consequences of the potentially harmful event (K factor). It can apply to all healthcare processes, including diagnostics, that do not produce the expected results and/or may cause harm to a patient [4].

As it is impossible to completely eradicate error in a complex organization such as the healthcare system, the chances of error and the consequences of error when it occurs must be minimized [5].

**Figure 1:**
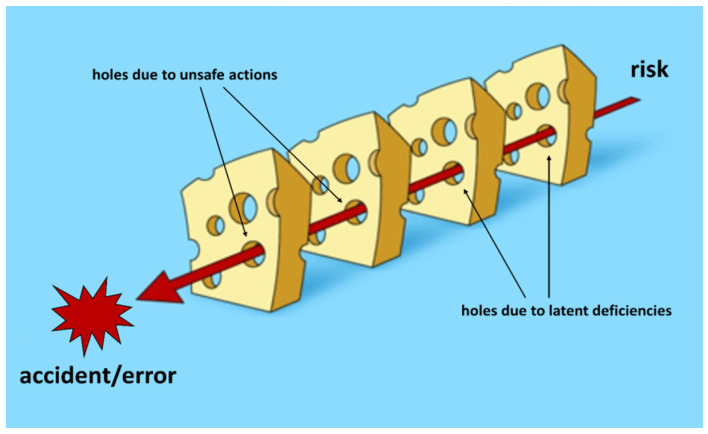
In the “Swiss cheese” model of error theory, defenses, barriers and guards may be breached by the trajectory of an accident. Modified from Reason [5].

According to Reason [5], errors are seen as consequences rather than causes, originating not so much, and not only, from human fallibility as from ‘upstream’ systemic factors, including recurring workplace error traps and the organizational processes that generate them. Countermeasures are based on the assumption that, although human error cannot be eliminated, it is nevertheless possible to identify flaws in a work process and raise the defenses of the system. When an error occurs, the important question is not who did something wrong, but how and why the defenses failed. Therefore, errors are caused by a succession of “favorable events” that have breached the defense mechanisms implemented (Figure 1).

**Figure 2:**
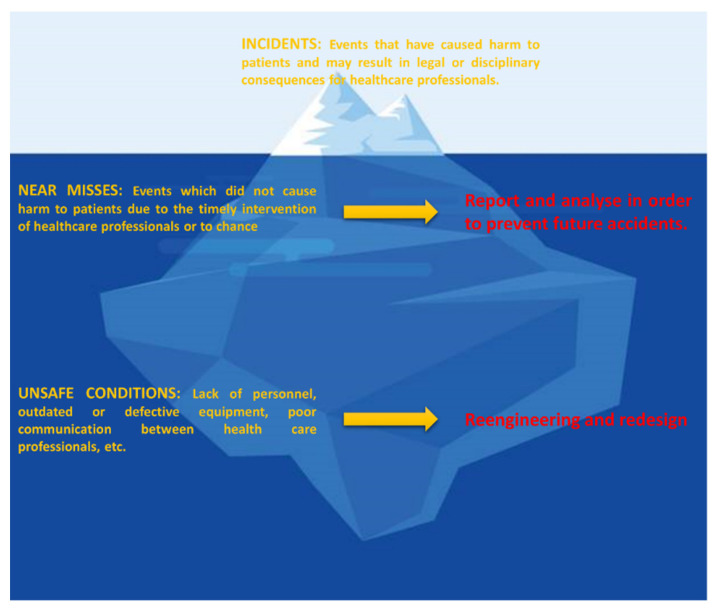
The iceberg model. Modified from the American Data Network [7].

The Iceberg model is a very simple but powerful way of representing the complexity of a system, in which what appears above the waterline is only the tip. Indeed, errors and incidents that emerge are actually just the tip of the iceberg, as many others (missed events) have not occurred simply because an audit prevented them (Figure 2) [6,7]. However, what lies beneath the surface offers a much deeper understanding of the phenomenon. The presence of near misses and unsafe conditions may reveal holes in the process that contribute to the occurrence of errors. People’s beliefs about how things work and the structures within which they operate may have a profound influence on their decision-making process. By bringing incidents to light and examining them through audits, an organization is forced to review its process design and eliminate any flaws that may have contributed to the incident.

## 2. The Skin Biopsy and Its Limitations

Clinical risk is intrinsic to all healthcare processes, including diagnostic procedures such as skin biopsies. Indeed, a skin biopsy is a complex process that goes well beyond merely removing a sample of skin. Maximizing the information provided by a skin biopsy necessitates fulfilling essential prerequisites: formulating a diagnostic hypothesis and/or a detailed morphological description of the clinical picture; selecting the appropriate biopsy technique and site; evaluating complementary and ancillary investigations; and, finally, integrating clinical and histopathological data.

**Figure 3:**
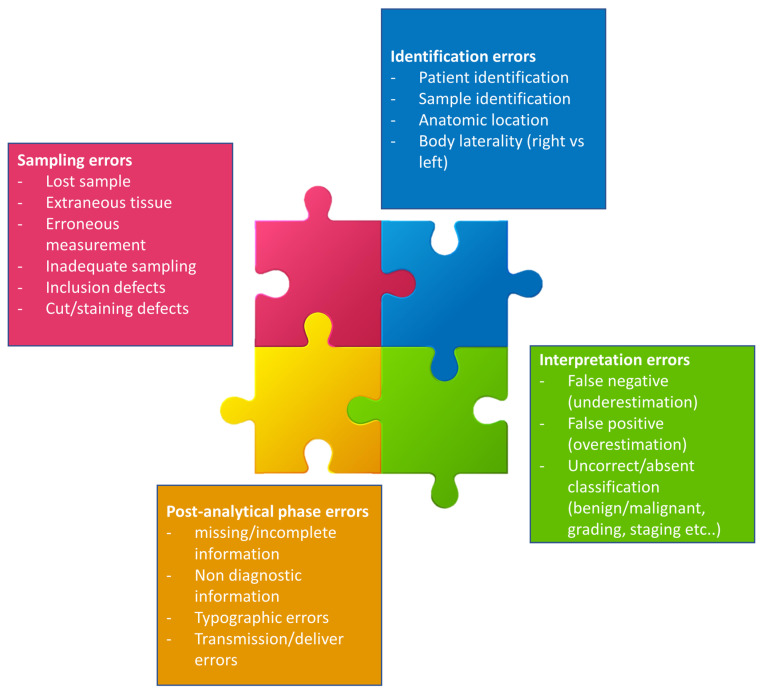
The process steps are strongly connected one to the other, and an error in one of them can negatively affect the outcome of the process. Modified from Zarbo R.J et al. [11].

There are several potential pitfalls that inevitably limit the diagnostic value of a skin biopsy and may lead to a misdiagnosis, adverse patient outcomes and increased healthcare costs. Indeed, errors may occur during the pre-analytical phases (sampling, identification, insufficient information, cutting and suboptimal inclusion), the analytical phases (interpretation) and/or the post-analytical phases (Figure 3) [8,9]. Each step in the skin biopsy care process relies on clear communication. Watson et al.’s study reported that pre-analytical errors accounted for 23% of medical errors in dermatology practice [10].

The pre-analytical phase encompasses all the procedures carried out before laboratory testing [12], where the dermatologist plays a pivotal role in its optimization. Dermatologists take pictures of the lesion before biopsy and select the most appropriate skin site for sampling with the aid of a dermatoscope to obtain the most sensitive and specific information. Moreover, a dermoscopy-guided biopsy is very useful for incisional biopsies of pigmented lesions. Digital videodermatoscopy may also be used to acquire images to correlate histopathologic findings with dermoscopy changes [12,13]. In excisional biopsy, lesional landmarks, corresponding to significant dermatoscopic elements, guide the pathologist’s choice of cuts in the selected region/s. However, if skin biopsies are carried out by non-dermatologists, the result may not only be poor-quality pathology reports but also ones that are not cost-effective.

Although providing the dermatopathologist with sufficient clinical information to work with might seem obvious, unfortunately, the necessary details are not always communicated. In fact, often incomplete, inaccurate, or even minimal (a code) information accompanies the sample, severely limiting the diagnostic abilities of the pathologist [14].

Health care providers’ ineffective communication and interaction significantly contribute to sentinel events and incidents [2,3]. It is estimated that diagnostic errors [4,5] occur at a rate of 10–20 percent, and may occur at any stage of the process, resulting in adverse outcomes for patients and a substantial increase in healthcare costs.

Generally, analytical errors are more likely to have a negative impact on patient care, with potentially serious consequences for the patient and the pathologist alike. Numerous literature reports have demonstrated that poor communication increases the clinical risk when it comes to interpreting skin biopsies [15,16,17]. According to a survey carried out by the Mayo Clinic in Minnesota, USA, nearly half of all pathologists spend at least 30 min every day gathering information from clinicians so as to be able to make a diagnosis, wasting time and resources and leading to dissatisfaction on both sides [15].

It is well known that the histopathologic diagnosis of melanocytic skin neoplasms is often a matter of considerable debate, even among experienced histopathologists. A multicentric study demonstrated that the histopathologic criteria in the diagnosis of melanocytic skin neoplasms can work as such, but the final diagnosis is a clinically aided interpretation [17]. Indeed, in this study, some histopathologic diagnoses were switched to benignity after studying images from clinically/dermoscopically atypical lesions. Therefore, clinical information, including the time of onset and evolution of a lesion, integrated with clinical and/or dermatoscopic images, may provide a higher diagnostic sensitivity and specificity of the clinical risk [18]. This approach may be particularly effective in the diagnosis of melanocytic lesions, where errors are frequently due to an underestimation of the lesions [19], descriptive or interlocutory reports and abuse of ambiguous diagnostic categories or acronyms, such as SAMPUS (Superficial Atypical Melanocytic Proliferations of Unknown Significance) and MELTUMP (MELanocytic Tumours of Uncertain Malignant Potential) [20].

## 3. Dermatopathology in Italy 

Until the end of the 20th century, Italian dermatologists carried out most of the dermatopathology services in dedicated laboratories, particularly in large and/or university dermatologic departments. One important advantage of this approach was that the pre-analytical, analytical, and post-analytical phases of skin biopsies were performed in the same place. This allowed for real-time clinical-pathological correlation whenever there was discordance with the clinical diagnosis or interpretive difficulties, and before the final report was drawn up, each “critical” case was discussed between the clinician (dermatologist) and the dermatopathologist during the patient’s scheduled check-up.

In 2000, the first Italian-accredited Training Centre for the International Committee of Dermatopathology (ICDP)/ International Board Certifying Examination—Diploma in Dermatopathology—was established in Turin, and it flourished for some years under the guidance of one of us (CT), with many dermatologists and pathologists from Europe applying for their residency training (http://www.icdermpath.org, accessed on 26 July 2011).

During the 1990s, Italy witnessed a progressive and inexorable centralization of the cutaneous histopathology laboratories (Regional Law No. 31 of 11 July 1997 on “Reorganization of the regional health service and its integration with the activities of social services”; Regional Council Resolution VII/3313 of 2001; Regional Council Resolution XI/772 of 2018) [21]. The economic crisis and the consequent strong pressure to reduce healthcare costs most likely also played a significant role in accelerating this process [22]. Unfortunately, little was achieved to the advantage of “efficiency” or the integration of those dermatologists who had acquired vast expertise in cutaneous histopathology. This led to a professional impoverishment of dermatology, both in terms of specialistic training and the quality of the care provided.

Most general pathologists were not trained in cutaneous pathology and, therefore, had to rely on second-level investigations, such as immunohistochemistry and/or genotypic studies, which were often not only of little use, but also sometimes even confusing, leading to delays in diagnosis/treatment and additional costs. The final result was a space-time break in the relationship between the physician and patient and a geometric enlargement of the clinic-laboratory interface, with an increase in complexity and problems involving safety and the quality of the healthcare provided [23].

Unfortunately, Italy was not the only European country where this happened. To mention but a few, this was also the case in France, Sweden and Germany [24].

## 4. Material and Methods

An internal analysis of pathology skin biopsy reports pertaining to a twelve-month period, i.e., from 1 January 2019 to 31 December 2019, was carried out so as to make an objective evaluation of the efficacy, critical aspects and impact they had on the patients’ outcomes in our Dermatology Department. 

Two main process indicators were selected: (1) the number of descriptive/non-diagnostic pathology reports, and (2) the number of discordant diagnoses, i.e., pathologic diagnoses that were in conflict with the clinical data. Discordances were weighted according to their potential impact on patient management and classified into three types: A, B or C. “Type A” reflected a discordance that had minimal or no impact on management, e.g., lichen simplex chronicus versus prurigo nodularis; “Type B” reflected discordance with a potential impact on management, but usually did not harm the patient, e.g., lichen simplex chronicus versus lichen planus; “Type C” reflected discordance with a significant impact on management and potential harm to the patient, for example, atopic dermatitis versus mycosis fungoides or nevus versus melanoma [25].

## 5. Results

A total of 2425 pathology skin biopsy reports were retrieved for the study period from our hospital’s digital archives. A total of 482/2425 biopsies (19.8%) were performed due to a clinical suspicion of an inflammatory or infectious dermatoses, while 1943/2425 (80.2%) were done to exclude skin neoplasms, and 607/1943 (31%) involved melanocytic lesions. 

A total of 426/2425 (17.6%) pathology reports were “descriptive”, i.e., they did not provide diagnostic interpretations. A total of 281/426 (66%) were reports on incisional biopsies of inflammatory dermatoses, 120/426 (28%) involved non-melanoma skin cancer biopsies and 25/426 (6%) were melanocytic lesions of uncertain nature. Moreover, there were 15 cases of pathologic diagnoses that were discordant with the clinical features. In some cases, two or more skin biopsies had been performed to resolve the conundrum. There were two cases of discordance type A, four of discordance type B and nine of discordance type C; ten cases involved inflammatory dermatoses and four skin neoplasms (Table 1).

As a detailed analysis of all these cases is beyond the scope of this paper, only two critical cases of neoplastic pathology and one case of inflammatory pathology (discordance type C) are described, where underestimation errors, combined with insufficient data and a lack of clinical-pathological correlation, led to high clinical risk for the patients.

### 5.1. Case 1

**Figure 4:**
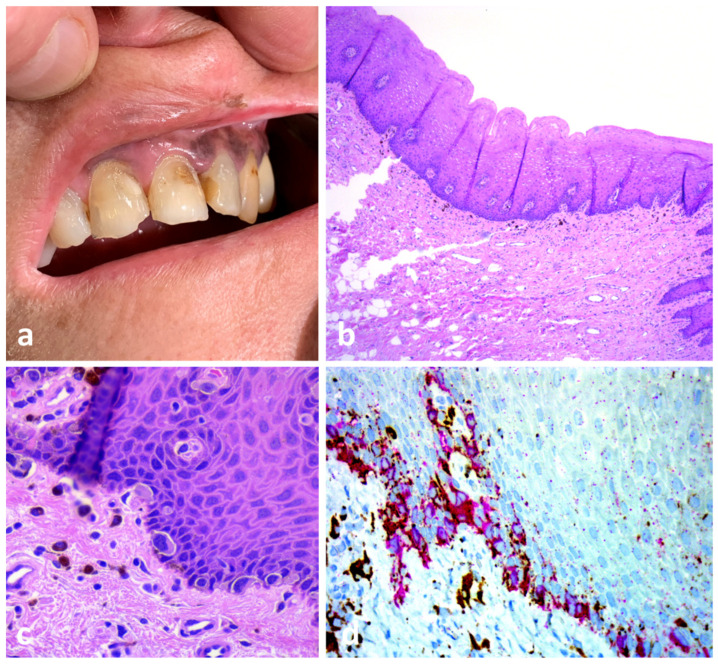
OMMIS misinterpreted as SAMPUS. (**a**) Upper lip melanoma recurrence with extension to the gingival arch. (**b**) A low-power view shows a seemingly normal gingival mucosa. (**c**) High magnification revealed an increased number of non-equidistant, large, atypical melanocytes along the basal layer. (**d**) Crowding of HMB-45 positive melanocytes with prominent dendrites along the basal layer.

A 56-year-old healthy woman presented with brownish pigmentation involving the left side of her upper lip, inner labial mucosa and adjacent gingiva (Figure 4a). Her clinical history revealed malignant lentigo of her upper left lip, which had been surgically removed 4 years before with “free margins”. A biopsy of the pigmentation on her upper gingiva was made. The histopathologic diagnosis was superficial atypical melanocytic proliferation of uncertain significance (SAMPUS). However, retrospective clinicopathologic correlation led to a diagnosis of oral mucosal melanoma in situ (Figure 4b–d).

Comment: Malignant melanoma remains the most contentious of all diagnoses in dermatopathology. The diagnosis of oral mucosal melanoma in situ (OMMIS) is challenging due to the subtle character of the pathologic changes. The usual presenting finding of mucosal melanoma is a lentiginous growth pattern of single cells along the interface region of squamous mucous membranes (mucosal lentiginous melanomas) [26]. In the early stage, nesting and pagetoid scattering into the epithelium are rare and relatively limited compared to superficial spreading melanoma and occur when the lesion is more advanced. Therefore, evaluating histopathologic findings along with clinical and macroscopic data and a close collaboration between the dermatologist and the pathologist are fundamental.

### 5.2. Case 2

**Figure 5:**
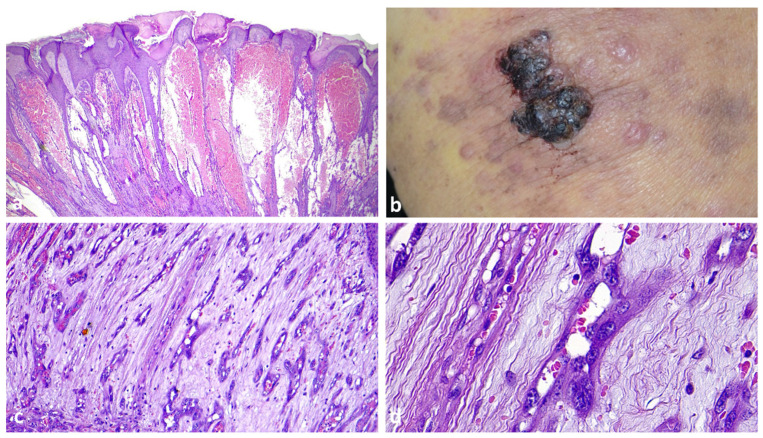
Cutaneous angiosarcoma misinterpreted as a pyogenic granuloma. (**a**) A scanning view showing an exophytic neoplasm with a pyogenic granuloma-like appearance. Remarkably, there is a lack of septation and lobulation. (**b**) A large violaceous plaque with ill-defined margins and a necrotic crust on the right thigh. (**c**) A proliferation of irregularly shaped anastomosing vascular channels within a loose mucinous stroma can be observed at the base of the lesion. (**d**) Hematic vessels are lined by severely atypical endothelial cells protruding into the lumen.

A 4 mm punch biopsy from a recent-onset cutaneous lesion of uncertain nature on the right thigh of a 93-year-old woman was sent to the pathology department for histopathologic examination. The pathologist made a diagnosis of pyogenic granuloma (Figure 5a). Clinicopathologic correlation prompted a review of the slides, and a retrospective definitive diagnosis of angiosarcoma was made (Figure 5b–d).

Comment: The early histological alterations of cutaneous angiosarcoma can be rather bland and may easily be mistaken for benign vascular tumors [27]. In this case, the exophytic and papillated lesion profile, with prominent and ectatic hematic vessels vaguely reminiscent of pyogenic granulomas, led the pathologist to make an erroneous decision not to examine the cytomorphologic details of the vascular proliferation at the bottom. Moreover, insufficient clinical data contributed to the diagnostic error. 

### 5.3. Case 3

**Figure 6:**
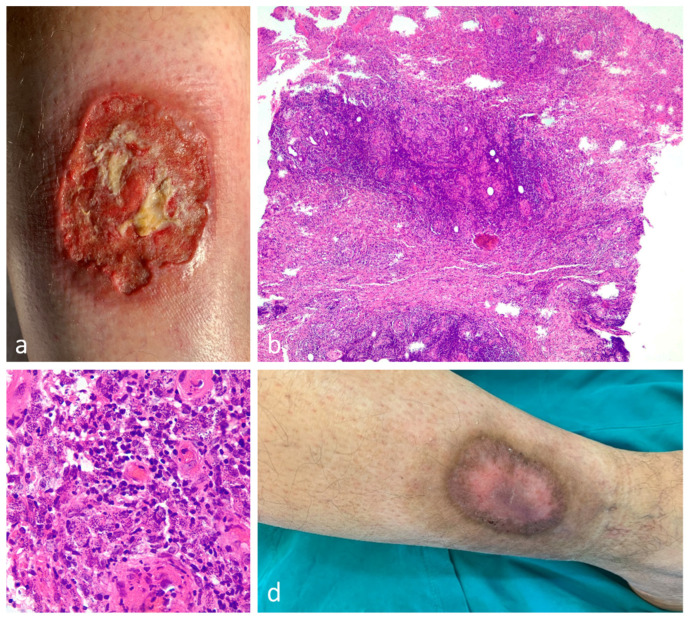
Ulcerative cutaneous leishmaniasis misinterpreted as pyoderma gangrenosum. (**a**) A large cutaneous ulcer with a bleeding, fibrinoid bed and an irregular, violaceous, undermined border. (**b**) A cutaneous ulceration with a highly vascularized dermis and diffuse inflammatory infiltrate. (**c**) High magnification showing Leishmania amastigotes within the macrophages. (**d**) The healed lesion after itraconazole treatment.

A 42-year-old healthy man developed an abraded lesion on his right leg after a minor trauma while traveling in southern Italy two years before admission. Shortly after, the lesion evolved into an ulcer that slowly enlarged, reaching its current size (Figure 6a). A biopsy was taken for suspicion of pyoderma gangrenosum, and the pathology report was consistent with this diagnosis. The patient was then treated with local and systemic immunosuppressive drugs, but the ulcer continued to grow. The slides were reviewed, and acute cutaneous leishmaniasis was diagnosed (Figure 6b,c). The patient was treated with itraconazole at 200 mg/day for 2 months with clinical cure of the lesion (Figure 6d).

Comment: Unusual skin ulcers frequently represent a diagnostic challenge. In this case, a history of trauma at the site of the ulceration supported the clinical diagnosis of pyoderma gangrenosum. However, this was misleading as it led the pathologist not to make a thorough examination of the tissue sample. Mechanical trauma and injuries have been sporadically described as triggers for the development of ulcerative cutaneous leishmaniasis in endemic areas. In this patient, the failure to respond to well-known and effective treatment options for pyoderma gangrenosum called this diagnosis into question [28,29].

## 6. The Need for the Project 

Nowadays, it is common practice in many European countries, such as Switzerland, Germany, Austria and Great Britain, to name but a few, for clinicians, pathologists and dermatopathologists to hold multidisciplinary meetings, especially when having to deal with pigmented lesions and melanoma. These meetings provide an opportunity to discuss and review all the clinical and anamnestic documentation as a group, including clinical and dermatoscopic images, where exchanges of opinions help clarify the pathologic changes [30]. A second opinion by a qualified pathologist or a board-certified dermatopathologist is crucial and often leads to important modifications being made in clinical management [31,32]. It would be nice to see these integration models become common practice between dermatologists and pathologists to guide and develop diagnostic pathways in dermatology, also in Italy.

Although limited to a relatively short period, the results of our preliminary analysis, should have sufficed to raise critical issues as to the process under investigation, i.e., the skin biopsy in a context lacking a clinical-pathological correlation. Indeed, pathologists are all too often provided with incomplete or inaccurate clinical information that hinders their diagnostic capabilities, which may lead to a clinical risk for the patient and have a negative effect on the cost/effectiveness ratio. According to a recent study, the estimated increase in the costs of treatment due to progression from stage 0 (melanoma in situ) to stages I, II and III range from about $4648 for melanoma in situ to $159,808 for stage IV melanoma [33].

In the project, rather than addressing all skin biopsies, the team will concentrate on selected complex cases of cancer pathology, inflammatory diseases and lymphoproliferative disorders. This can be accomplished by establishing scheduled, ongoing meetings where challenging clinicopathologic cases are presented and collegially discussed. Providing more complete clinical data, such as detailed anamnesis and clinical and dermoscopic findings, at these meetings and collegial discussions can help solve interpretative doubts, reduce histopathologic differential diagnoses and reach a collegial discharge diagnosis. In this way, diagnostic errors and the use of non-diagnostic pathologic categories can be reduced (e.g., SAMPUS, MELTUMP).

The meetings should be held on a regular basis at a fixed time, date and location. The location should be suitable, i.e., fully equipped with multihead microscopes, projectors and personal computers, and large enough to accommodate all participants.

Minutes signed by the group coordinator are to be prepared at the end of each session, reporting the list of participants, the cases/patients discussed and the final discharge diagnosis. The pathology report is to be signed whenever possible by both the referring pathologist and the clinical dermatopathologist. Alternatively, the statement “case discussed by the multidisciplinary team” should be added after the diagnosis.

All documentation concerning the cases is to be stored in a folder within the organization’s computer network, which may be shared remotely by all members of the group and accessed whenever necessary. Residents in dermatology and pathology will also be invited to join the team.

Multidisciplinary teams are examples of healthcare matrix organizations (ones in which there is dual or multiple managerial accountability and responsibility), which bring together skills, knowledge and attitudes (core competencies) divided into different functions to achieve healthcare objectives. The members of the healthcare matrix team are to report to both the project manager and the department head. This type of organization has several advantages, including the dissemination and sharing of knowledge and skills amongst professionals who, despite belonging to different functional departments, share a common objective, i.e., correctly diagnosing and treating skin diseases. Furthermore, a matrix organization would allow for the selection of team members based on their ability, suitability and availability. The duties and responsibilities of the project manager and department head must be clearly defined, so as to avoid confusion due to a dual reporting line for the healthcare personnel involved.

## 7. Resistance to Change and Innovation

Change is a universal phenomenon that impacts organizations of all types and times [34]. Therefore, there are numerous factors that may hinder the implementation of the project, including the presence of an organizational culture and internal power structure, the difficulty in accepting change due to psychological mechanisms relating to beliefs, habits, practices, automatisms, social context and/or group dynamics, as well as the fear of losing identification with one’s job description and self-perception based on the type of work performed. In order to limit resistance to change, context analysis and the involvement of professionals through effective communication of the reasons for the initiative, reconnaissance of inputs and support activities are crucial from the very earliest stages of the definition of a project, and this is the responsibility of the hospital management [34,35,36,37]. Moreover, the COVID-19 pandemic has intensified the complexity of the dermatological landscape, with clinics and hospitals facing reduced services, prioritization of urgent cases and closures due to staffing shortages or resource diversion. Patient reluctance to visit healthcare facilities for fear of exposure has also led to delayed diagnoses and treatment, exacerbating the dermatology backlog [38,39].

## 8. Preliminary Results

Our internal analysis of the sensitivity and specificity of skin disorder pathology reports over the study period led to the setting up of a pilot project on 1 January 2022. A group of dermatologists and general pathologists from the Fondazione IRCCS Policlinico San Matteo, a university hospital in Pavia, met twice a month to discuss the histopathological specimens of selected cases with ongoing diagnoses. The project was run by personnel from within the organization, and the resources available were taken advantage of, including a Board-certified dermatopathologists (CT) with clinical expertise. A total of 163 cases were discussed by the members. A final discharge diagnosis was reached in 142/163 cases (87.1%), and the patients were treated accordingly. A total of 151/163 cases involved inflammatory dermatoses, while the remaining cases were neoplastic dermatoses. Whenever a definitive diagnostic report was not reached, the cases were inflammatory dermatoses. The neoplastic cases included two basal cell carcinomas, one squamous cell carcinoma, one trichilemmoma and two cutaneous lymphoproliferative disorders. These findings emphasize the highly challenging and unique nature of inflammatory skin pathology. This is evident not only because the majority of cases brought to the multidisciplinary team were inflammatory dermatoses, but also because the only cases that remained with descriptive reports were also inflammatory dermatoses. These preliminary encouraging results indicate that a multidisciplinary approach to the histopathologic diagnosis of skin diseases may provide several benefits to both members and the organization as a whole:

▪ Cognitive: allowing for a holistic understanding of inflammatory and neoplastic cutaneous diseases, expanding individual perspectives and responsibilities;
▪ Motivational: enhancing motivation by enabling shared decision-making;
▪ Relational: fostering enhanced empathy, a sense of belonging, and mutual recognition among different professionals;
▪ Organizational: improving integration within an organization by promoting the sharing of knowledge, languages, methods and goals.

An analysis of the strengths, weaknesses, opportunities and threats (SWOT) of our project is provided in Table 2.

The most significant limitation of the present proposal is the shortage of professionals with expertise in both dermatopathology and dermatology. Indeed, during the last three decades, at least in Italy, the limited opportunities for hands-on practice and career advancement within public healthcare organizations and/or university hospitals have led most residents and young dermatologists not to invest in cutaneous histopathology, but rather to dedicate their time and resources to other areas of dermatology. However, we are of the opinion that, if the present project were to be implemented on a large scale, both in Italy and elsewhere, then the demand for skilled clinically or pathologically trained dermatopathologists would increase.

That said, complying with the specific Italian rules/regulations governing the practice of medicine is a must. These rules/regulations outline the types of medical activities the various categories of physicians are allowed to perform and may vary depending on the context the activity is carried out in, e.g., whether it is being done in a private or public setting. There are no restrictions on the types of medical activities that a licensed medical doctor can perform in a private practice, with the exception of radiology and resuscitation. Conversely, the norms are more complex within the Italian National Health System, and, according to Presidential Decree 483/1997 (Regulations on competition tender for the managerial staff of the National Health Service), it is necessary to have a Diploma of Specialty to do so [40]. Meaning that, so as to be allowed to report on a skin biopsy or carry out any other activity pertinent to pathology within the Italian NHS, even if limited to cutaneous pathology, a physician must be specialized in pathology. 

Noteworthy is the fact that, in Europe, both pathologists and dermatologists with adequate training and experience in dermatopathology can obtain specialized qualifications offered by the Royal College of Pathology and/or the European Union of Medical Specialists). In Switzerland, dermatology and venereology specialists wishing to specialize in dermatopathology must complete a 24-month training program, which includes 12 months of pathology and 12 months of dermatopathology. Candidates must have previously qualified as specialists in dermatology and venereology. During the training, the candidate must evaluate only skin biopsies and independently evaluate a total of 6000 surgical samples, which include skin biopsies. The candidate must also participate in six postgraduate training events in dermatopathology and publish a scientific paper as the first author in the field of dermatopathology [41]. In fact, dermatopathology is an integral part of the pathology residency training program, and pathology residents are required to acquire expertise in dermatopathology if they are to obtain their specialization. In addition to providing training and guidance to general pathologists rotating in dermatopathology, dermatopathologists also provide valuable assistance to general pathologists, especially when it comes to diagnosing inflammatory skin diseases, as general pathologists often lack clinical dermatology training and have limited experience in this field. That is why general pathologists are included in clinico-pathological correlation meetings.

**Figure 7:**
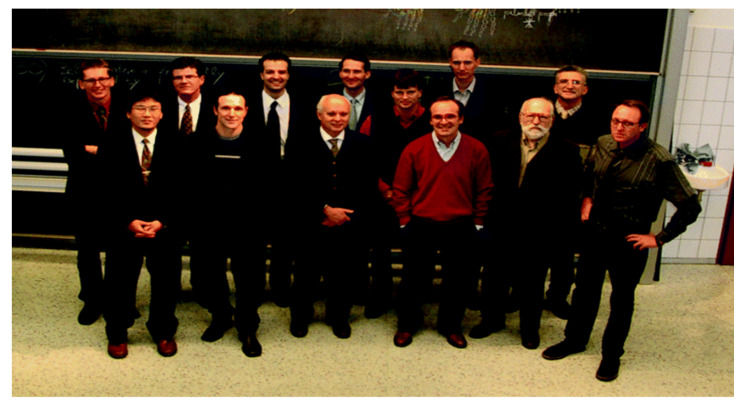
The first certifying examination in dermatopathology. Candidates from Europe and Asia who went to Frankfurt to sit the examination. Reproduced from Kerl H. et al. with permission from Wiley [42].

The first Certifying Examination in Dermatopathology (International Diploma of Dermatopathology), organized by the International Board Certification in Dermatopathology, was held in Frankfurt in 2003, with both pathologists and dermatologists sitting for it (Figure 7) [42]. The document The Management of Dermatopathology, developed by the European Dermatology Forum Subcommittee in 2014, states that once a dermatologist passes the exam, this would mean recognition of competence in dermatopathology [43].

## 9. Conclusions 

A cost–benefit analysis of this project demonstrated that the first beneficiary is the patient, who is provided with a service (the biopsy) that leads to an outcome in terms of health value that is potentially maximized. The second beneficiary is the dermatologist, who performs a skin biopsy to resolve a diagnostic doubt and treat the patient appropriately. The final beneficiary is the pathologist, who can reduce the risk of reporting errors thanks to adequate clinical-pathologic correlation.

Although the increase the workload for those involved in the project was only a few hours per week, it is still important that this additional effort be recognized by the organization. Moreover, competence, which includes skills, knowledge and motivation, is an important factor in the success of the project.

The Weggeman model, as revised by Goldschmidt [44], describes competence as a combination of skills, knowledge, self-esteem, attitudes and motivation. A modern healthcare organization cannot rely solely on efficiency to determine its success, as competence is equally important in producing excellent results. While a low implementation cost may encourage early adoption of the project by the organization, a no-change alternative may also generate negative consequences. 

The road to harmonization among medical disciplines is quite long, and the future perspectives are not based on an unrealistic unification of related content and practices, but rather on developing operational standards and training pathways to reduce variability among subspecialties, enhance processes and foster expertise development. Reengineering processes and prioritizing skills over rigid contractual positions may allow for the introduction of persons with specific competences, favoring harmonization between medical specialties, which is an essential element in providing appropriate care; indeed, this project was inspired by these principles. [45]. 

## Figures and Tables

**Table 1:** Classification of clinicopathological discordances in 15 patients.

Case Number	Age (Years)	Gender (M/F)	Diagnostic Category	Biopsy Site	Clinical Diagnosis	Histopathological Diagnosis	Discordance Type (A/B/C)	Final Discharge Diagnosis
1	71	F	Inflammatory	Trunk (folds)	Interstitial granulomatous dermatitis	Chronic dermatitis	B	Interstitial granulomatous dermatitis
2	69	F	Inflammatory	Trunk	PRP	Psoriasis	B	PRP
3	56	F	Inflammatory	Trunk	Morphea	Chronic dermatitis	C	Morphea
4	72	M	Inflammatory	Upper limb	AGEP	Impetigo	C	AGEP
5	56	F	Neoplastic	Oral mucosa	Mucosal melanoma	SAMPUS	C	Mucosal melanoma
6	93	F	Neoplastic	Lower limb	Angiosarcoma	Pyogenic granuloma	C	Angiosarcoma
7	68	F	Inflammatory	Upper limb	Granuloma annulare	Chronic dermatitis	B	Granuloma annulare
8	86	M	Neoplastic	Ear	Chondrodermatitis nodularis	Squamous cell carcinoma	C	Chondrodermatitis nodularis
9	42	M	Inflammatory	Lower limb	Chronic ulcer	Pyoderma gangrenosum	C	Leishmaniasis
10	58	M	Inflammatory	Face	Mycosis fungoides	Spongiotic/atopic dermatitis	C	Mycosis fungoides
11	68	M	Inflammatory	Upper limb	Eosinophilic fasciitis	Morphea	A	Eosinophilic fasciitis
12	46	M	Neoplastic	Upper limb	Eccrine poroma	Clonal seborrheic keratosis	A	Eccrine poroma
13	90	M	Inflammatory	Trunk	Erythroderma. CTCL	Spongiotic dermatitis	C	Mycosis fungoides
14	48	F	Inflammatory	Trunk	Photoaggravated dermatosis	Psoriasiform dermatitis	B	Subacute cutaneous lupus erythematosus
15	55	F	Inflammatory	Trunk	Mycosis fungoides	T-cell pseudolymphoma	C	Mycosis fungoides

**Table 2:** The SWOT analysis of the project.

**Strengths**	**Weaknesses**
▪ Well-established international models; ▪ Enhanced efficiency and effectiveness of the reporting process (evidence-based, European and/UEMS guidelines); ▪ Resource integration, professional development and relationship building (teams); ▪ Dermatologists interested in developing dermatopathology skills.	▪ Regulatory limitations apply to the reporting of histological examinations without a specialization in pathological anatomy; ▪ Even if the clinicians have a Board of Dermatopathology qualification, they are not legally allowed to sign out pathology reports in public hospitals; ▪ Dermatology does not carry a high political-contractual weight in many hospitals; ▪ Exiguity of dermatologists with the Board Certification leads to an even lower contractual weight.
**Opportunities**	**Threats**
▪ Definition of dermatopathology training standards through ministerial regulation; ▪ Development of legislation to legitimize the dermatologist with the International Board in the reporting of histopathological examinations; ▪ Better strategic positioning for dermatology and the organization; ▪ Enhancing the qualifications of the dermatologists; ▪ Developing professional paths (Masters and UEMS-accredited training centers) and international recognition of UEMS-accredited training centers; ▪ Repositioning and upgrading of dermatology at a local, national and international level.	▪ Resistance to change and innovation; ▪ Politically influential stakeholders (Pathologists’ Societies); ▪ Unfavorable reactions from pathologists and their societies; ▪ Dermatopathology skills are being acquired by pathologists as well as clinical skills (competitors).

## Data Availability

Data are in the article.
